# R/UAStools::plotshpcreate: Create Multi-Polygon Shapefiles for Extraction of Research Plot Scale Agriculture Remote Sensing Data

**DOI:** 10.3389/fpls.2020.511768

**Published:** 2020-09-30

**Authors:** Steven L. Anderson, Seth C. Murray

**Affiliations:** Department of Soil and Crop Sciences, Texas A&M University, College Station, TX, United States

**Keywords:** shapefiles, open source software, small plot, agricultural, GIS

## Abstract

Agricultural researchers are embracing remote sensing tools to phenotype and monitor agriculture crops. Specifically, large quantities of data are now being collected on small plot research studies using Unoccupied Aerial Systems (UAS, aka drones), ground systems, or other technologies but data processing and analysis lags behind. One major contributor to current data processing bottlenecks has been the lack of publicly available software tools tailored towards remote sensing of small plots and usability for researchers inexperienced in remote sensing. To address these needs we created plot shapefile maker (R/UAS::plotshpcreate): an open source R function which rapidly creates ESRI polygon shapefiles to the desired dimensions of individual agriculture research plots areas of interest and associates plot specific information. Plotshpcreate was developed to utilize inputs containing experimental design, field orientation, and plot dimensions for easily creating a multi-polygon shapefile of an entire small plot experiment. Output shapefiles are based on the user inputs geolocation of the research field ensuring accurate overlay of polygons often without manual user adjustment. The output shapefile is useful in GIS software to extract plot level data tracing back to the unique IDs of the experimental plots. Plotshpcreate is available on GitHub (https://github.com/andersst91/UAStools).

## Introduction

Remote sensing platforms geared towards automated high-throughput crop monitoring have become important tools with potential to drive gains in crop improvement and management (Araus et al., 2018). Although curating sensor information/images has become somewhat run-of-the-mill, especially for remote sensing specialists, processing sensor information into informative data for decision making remains a tedious, time consuming, and challenging process (Shakoor et al., 2017; Shakoor et al., 2019). Aside from the processing/calibration of sensor datasets, reducing dataset dimensionality is a critical step in facilitating the ability to make actionable decisions. In plot-based agriculture research programs this requires the creation of individual areas of interest (AOI) for each research entry/treatment of interest. These AOIs are used to extract plot level information, such as the plant height, canopy cover, or vegetation index of a specific plot containing an individual genotype or experimental treatment. When the number of plots is small (<50), little effort is required and shapefiles containing AOIs can be manually drawn. However, for large plant breeding or genetics programs hundred to multiple thousands of plots, AOI may be needed and unique identifying information with consistent and repeatable labeling is needed for each AOI.

There are several features that are needed to make plot extraction from GIS software efficient, even for novices. (i) The ability to rapidly create a grid of polygons to be overlayed on plots in the proper rotation for any mosaic. (ii) The ability to easily incorporate the experimental design using tabular information with attributes, such as, unique plot IDs for each polygon. (iii) An option for buffering (i.e., a reduced representation of the plot polygon) to exclude areas of bare soil (e.g., walkways/alley) and reduce plot overlap when an orthomosaic has some distortion. (iv) Free and open source availability that allows all researchers to use the same tool without proprietary software.

Tools available to rapidly create AOI polygons for large scale small plot trials (> 100 of plots) are limited (**Table 1**), or unknown to the user community. ArcGIS (ESRI Development Team, 2019) and QGIS (QGIS Development Team, 2019) utilize a fishnet approach to create a regular gridded rectangle, although unique identifiers must manually be assigned to each polygon. Unique ID assignment is further complicated due to the left-to-right, top-to-bottom grid creation rather than the bottom-to-top, serpentine design commonly used in small plot design. ArcGIS and QGIS require identification of a four-point coordinate system to properly orient gridded polygons to the field-plot offset from north-south orientation. Plot Phenix (Progeny Development Team, 2019) “grid” functionality, a commercial software, resolves this issue through manual, point and click identification of corner plots, automated polygon centering, and a vast array of options to optimize polygon size, rotation, buffer, stagger, and subsetting. R/FieldimageR::fieldshape (Matias et al., 2020) and ImageBreed (Morales et al., 2020) “Generate Polygon Template” are open source software that create plot polygons based on manual, point and click identification of polygon grid corners in combination with total column and row counts. R/FieldimageR and ImageBreed link polygons back to unique IDs and plot design, but lack buffering functionality. Additionally, they provide plot, and image rotation capabilities are as separate functions/steps. Although several softwares are beginning to provide automated polygon gridding functions tailored to small agricultural research plots there is still a need for an open source resource that incorporates (i) plot orientation, (ii) experimental design, (iii) automated attribute table with unique plot ID, and (iv) plot buffering.

**Table 1:** Available software which can create gridded multi-polygon shapefiles.

Software	Function name	Connects unique ID	Utilized plot design	Automated buffer zone	Automated Rotation	Open Source
ArcGIS	Create Fishnet	F	F	F	T	F
ImageBreed	Generate Polygon Template	T	T	F	F	T
Plot Phenix	Grid	F	T	T	T	F
QGIS	Vector Grid	F	F	F	T	T
R/FIELDimageR	fieldShape	T	T	F	F	T
R/UAStools	plotshpcreate	T	T	T	T	T

## Implementation

**Figure 1:**
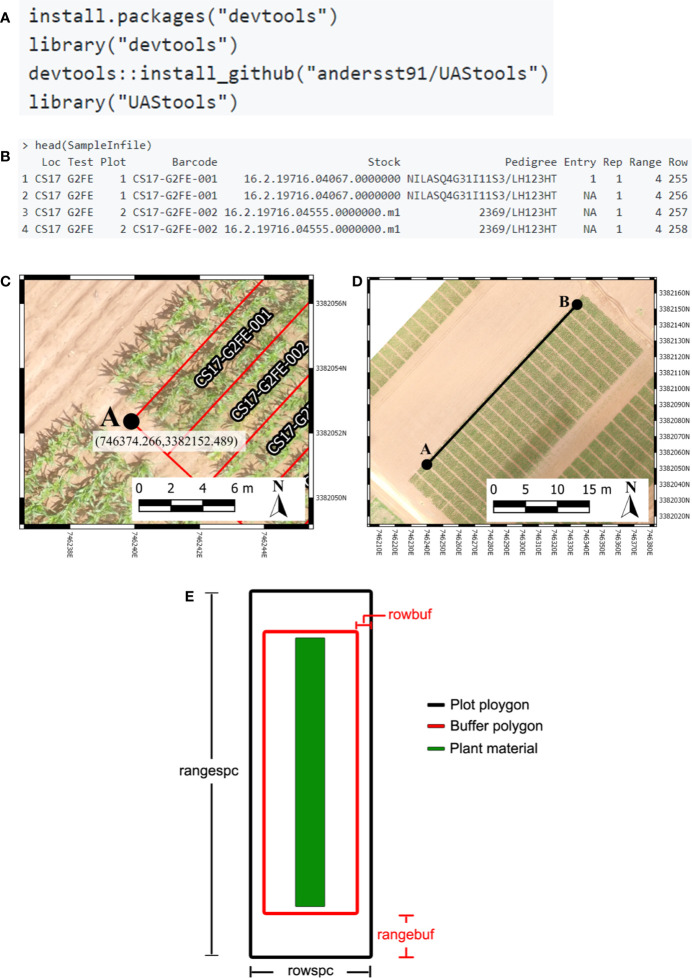
**(A)** Executable lines necessary to load UAStools into the R environment. **(B)** Example of common data structure used as the input file for plotshpcreate. **(C)** Demonstrates the localization of an “A” point in reference to the first plot polygon of respective experimental design matrix (front, left corner of the fist plot if reading a book from bottom to top, left to right). **(D)** Visual representation of A-B line. **(E)** Diagram demonstrating the plot (black) and buffer (red) polygon spacing input parameter. Portions of this figure were adopted from Anderson et al. (2019).

R/UAStools::plotshpcreate (**File S1**) is implemented as a software package function of R (**Figure 1A**), which constructs a multi-polygon shapefile (.shp) of a research trial, with individual polygons defining specific research field plots. Plotshpcreate has two dependency packages (R/sp (Pebesma and Bivand, 2005; Bivand et al., 2013) and R/rgdal (Bivand et al., 2019)) and is recommended to be executed on the most current version of R. Plotshpcreate has three main argument inputs (i) seed preparation and experimental design data frame (**Figure 1B**), (ii) A-B line coordinates (**Figures 1C, D**), and (iii) plot and buffer dimensions (**Figure 1E**). Output files include a multi-polygon ESRI shapefile using overall plot dimension and a multi-polygon ESRI shapefile using buffer plot dimension. Optional outputs include visual representations of shapefile for rapid accuracy assessment. UAStools can be loaded into the R environment using the devtools package (**Figure 1A**). Example scripts can be found using (i) “?plotshpcreate” command in R, (ii) the github wiki page (https://github.com/andersst91/UAStools/wiki/plotshpcreate.R), and (iii) example pipeline scripts (https://github.com/UFResearchComputing/PlantSci_BigData/blob/master/Workshop/UF_PSS_Script_v3.R).

### Required Inputs

#### Seed Preparation and Experimental Design Data Frame

The infile (e.g., R/View(SampleInfile); **Figure 1B**) for plotshpcreate.R requires four columns matching the quoted column names below (additional columns are permitted but won’t be utilized): (i) “Plot”: The number of each plot (numeric); (ii) “Barcode”: A unique identifier for each plot (character); (iii) “Range”: The range (also referred to as row in non-furrow irrigated agriculture systems and reflects the rows of your plot design matrix) number of each plot in the plot design matrix (numeric); and (iv) “Row”: The row (also called column in non-furrow irrigated agriculture systems) number of each plot in the plot design matrix (numeric). Barcodes must be unique across all observations if nrowplot=1 (i.e., if every observation of the infile has a unique barcode use nrowplot==1). Repeated barcodes and plot numbers if there are multi-row plots as the plotshpcreate function accounts for this redundancy within the function. Barcodes must be identical across adjacent rows in a plot if trial consists of multi-row plots. An example from the barcode system we typically use is “CS17-G2FE-018” where “CS” denotes the location, “17” denotes the year, “G2FE” denotes a trial in this location year and “018” denotes the 018^th^ plot within this trial. A sample dataset has been provided with R/UAStools and is defined in R as “SampleInfile” when UAStools is loaded *via* library(“UAStools”) command.

#### A-B Line Coordinates

Plotshpcreate was developed for Universal Transverse Mercator (UTM) coordinates. Convert to UTM before attempting to use plotshpcreate using a projection transformation tool (i.e., R/rgdal::project). Plotshpcreate builds plot polygons based on the “A” point (**Figure 1C**) as a reference and utilized the plot locations in the rectangular grid (Range, Row) of the plot design matrix to calculate the appropriate geo-locations for the polygon corners. The location of “A” is specific, and must lie at the front, left corner of the first plot of respective experimental design matrix (front, left corner of first plot if reading a book from bottom to top, left to right). More specifically, within the middle of the preceding alley and in the middle of the inter row space to the left of the first plot (**Figure 1B**). The B point is less specific but should be place in the same inter-row space to accurately capture the exact angle (i.e., deviation from South/North orientation) of the field (**Figure 1C**).

The best method for the A-B line development is using the geo-rectified orthomosaic of interest, alternatively a high-confidence handheld real-time kinematic (RTK) GPS on a pole to ensure an accurate A-B line in the field. A and B points can be identified using existing R packages (i.e., R/raster::plotRGB along with R/graphics::locator), but we recommend using QGIS (or equivalent software) due to improved resolution for identification of UTM point coordinates. If many temporal orthomosaics will be used throughout the season, one of these with high accuracy (e.g., GCP error) and low distortion can be used to develop plotshpcreate and subsequently applied to all other timepoints. Although use of a single shapefile across multiple orthomosaics is ideal, the user should be aware that error/inconsistencies in image stitching, as well as variances in orthorectification efficiency and accuracy could result in the inaccuracy of shapefile location when used to extract data from orthomosaics other than the reference mosaic. Visual accuracy checks are the simplest way to assess such accuracy issues and identify data sets that may need a dataset-specific shapefile. Use of plotshpcreate.R is possible with orthomosaics lacking geo-rectification, but requires the user to manually identify A and B points within each orthomosaic using a GIS software such as QGIS, ArcGIS, or R/rgdal. Users can loop plotshpcreate.R creating multi-polygon shapefiles for each unique non-georeferenced orthomosaic.

#### Plot and Buffer Dimensions

There are four polygon dimension arguments that can be specified to accurately create the proper plot dimensions and buffer dimensions desired (**Figure 1E**). Row (i.e., column) spacing (rowspc) spacing of a single row is set to 0.76 m in reference to the row spacing, by default. Range (i.e., row) spacing (rangespc) refers to the total plot length including half alley distance on either side of the plot (default: 7.62 m). Row buffer spacing (rowbuf) is the distance removed from both sides of rowspc to create a buffer zone between plots boundaries (default is 0.03 m). Range buffer spacing (rangebuf) is the distance removed from both sides of rangespc to create a buffer zone between plots boundaries (default is 0.61 m). As an example, if alleys are 1.22 m rangebuf they should be set to 0.61 m to remove 0.61 m from both ends of the polygon. These settings all must be changed for each researchers plots sizes, any default will almost never fit any other research study.

### Optional Functionality Arguments

We have designed plotshpcreate to have several useful functionalities that dictate how plot polygons can be created (**Table 2**). Plotshpcreate was developed based on a common style of seed preparation input files, meaning that if a plot consists of multiple planted rows, the input file must contain a row of data for each plot of the design/layout matrix (i.e., every range x row combination) with the same unique ID. There are ways to overcome this by adjusting plot dimensions and input file, but we will not discuss those methods.

**Table 2:** Gallery of plotshpcreate input parameters.

Parameter	Default	Options	Description
A	NULL	User	Numeric vector of UTM coordinates (Easting, Northing) of “A” point.
B	NULL	User	Numeric vector of UTM coordinates (Easting, Northing) of “B” point.
UTMzone	NULL	User	Character parameter defining UTM zone number. Default will result in an coordinate reference system of “NA”.
Hemisphere	“N”	User	Character parameter that designates the Northern “N” or Southern “S” Hemisphere.
infile	NULL	User	Data frame containing seed preparation file and experimental design
outfile	NULL	User	Character assignment to define output file names.
nrowplot	1	>0	Number of adjacent rows that constitute a plot/unique ID
multirowind	FALSE	Logical	Logic parameter that indicates if adjacent plot rows should be combined and treated as a single plot shapefile and unique identifier.
rowspc	2.5	>0	Row (i.e., column) spacing of a single row.
rowbuf	0.1	≥0	Distance removed from both sides of rowspc to create a buffer zone between plot boundaries.
rangespc	25	>0	Range (i.e., row) spacing of a single row.
rangebuf	2	≥0	Distance removed from both sides of rangespc to create a buffer zone between plots boundaries.
stagger	NULL	User	Numeric vector c(i, j, k) of length three defining [i] row where staggers starts, [j] rows sowed by planter in a single pass, and [k] stagger offset distance from A point.
plotsubset	0	≥0	Defines how many adjacent rows should be excluded from either side of the plot.
field	NULL	User	Character vector to indicate the trial the shapefile is being developed for. Example: CS17-G2FE
unit	feet	feet and meter	Character vector that the unit of measure for the polygon dimensions.
SquarePlot	TRUE	Logical	Logic parameter to indicate if PDF file is desired for visualization of non rotated polygons.
RotatePlot	TRUE	Logical	Logic parameter to indicate if PDF file is desired for visualization of rotated polygons.

**Figure 2:**
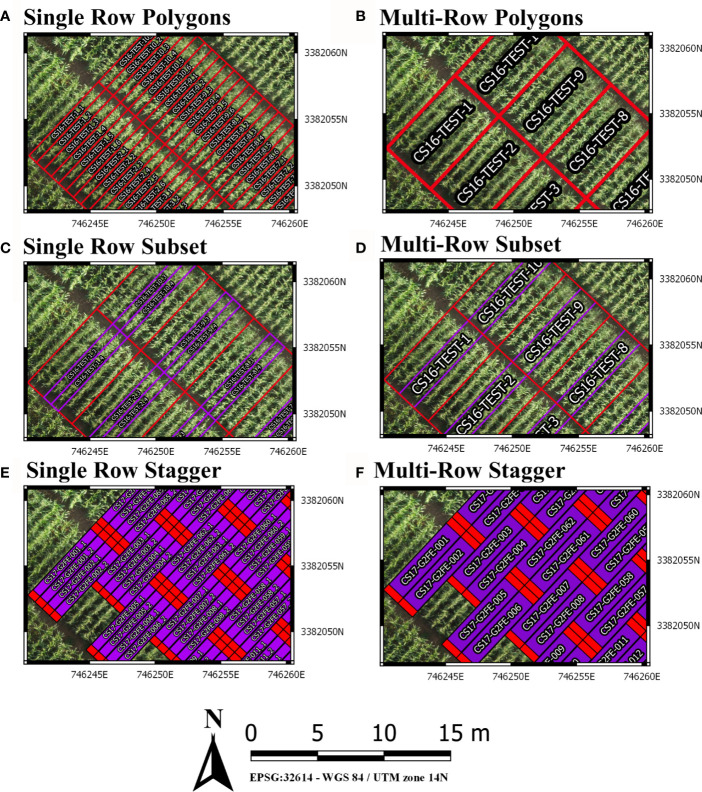
**(A)** Polygons Created for each individual row of a six row plot. **(B)** Single polygons created for each plot merging the adjacent rows of each plot. **(C)** Sub-setting out the middle two rows (purple) of a six row plot (red). **(D)** Sub-Setting out the middle two rows and merging them to a single polygon (purple) of a six row plot (red). **(E)** Staggering individual row plot polygons to adjust for staggered planting. **(F)** Staggering merged two row plot polygons to adjust for staggered planting.

The default arguments assume single row/range plots (nrowplot=1) and a unique barcode for each row of the input file (equivalent to **Figure 2A**). It is common to have multiple adjacent rows plots where researchers desire a single measurement representing the combined rows. Plotshpcreate combines multi-row plots (**Figure 2B**) based on matching barcodes by defining the number of rows a plot contains (nrowplot=“n”) and telling plotshpcreate to combine the rows (multirowind=F). Plotshpcreate can create single polygons of each row plot of a multi-row plot (**Figure 2A**), adding an index to each Unique ID in order to identify the data of the multirow plot from left to right (e.g., left row: CS17-G2F-018_1, right row: CS17-G2F-018_2, etc.). Individual row polygons of a multi-row plot can be created with the arguments multirowind=T and defining the number of rows a plot contains (nrowplot=“n”). Multirow plots with rows extracted individually in this way can be averaged after extraction or during analysis. However, while a two-row plot (for example) will double the number of observations, these will not be independent, and caution should be used in interpretation of degrees of freedom.

It is common in advanced yield trials to collect data from interior rows of a multi-row plot to factor out neighboring plot competition. Plotshpcreate has a built in sub setting functionally (plotsubset=“n”) to create polygons of those specific AOIs. The plotsubset argument works by removing “n” rows from either side of the multi-row plot and returns the remaining inter rows and can be used in combination with “multirowind” and “nrowplot” arguments (**Figures 2C, D**). For example, with a six row plot set “plotsubset=2”, plotshpcreate will return the inner two rows of the plot removing two rows from adjacent sides. Alternatively, all six individual plots could be extracted and the outer four discarded, however this would result in a threefold larger file taking additional time to extract and analyze, and the two inner rows would still need to be averaged in some appropriate way.

**Figure 3:**
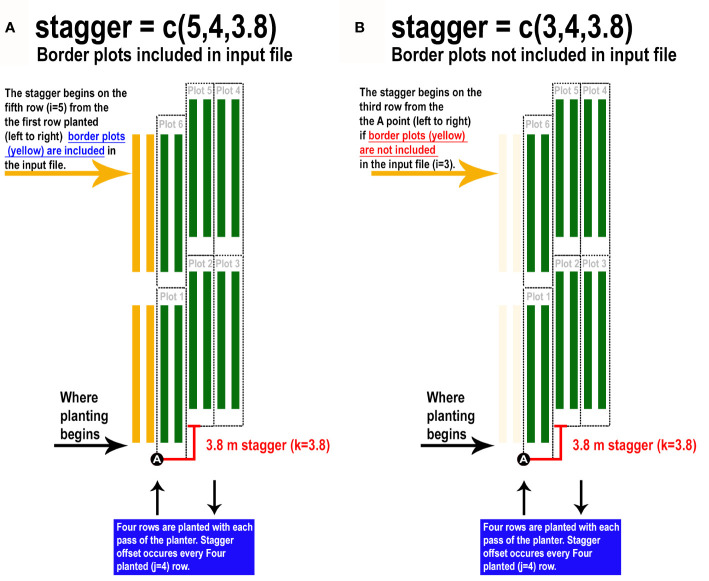
Illustration demonstrating how to properly implement the “stagger” argument of plotshpcreate if **(A)** border plots are incorporated withing the input file design matrix or **(B)** border plots are not incorporated withing the input file design matrix.

Furthermore, plotshpcreate can adjust polygon geolocation based on a consistent staggering of plot plantings caused by GIS or tripping issues (**Figures 2E, F**). Plotshpcreate adjusts row and range numbering to begin at one based on the input variable (i.e., if the minimum row number is three it will be adjusted to one, four adjusted to two, and so forth). This is important to remember when utilizing stagger whether field border rows are incorporated within your input file matrix. Plotshpcreate can create staggered plots grids with an input vector (stagger=c(i,j,k)) describing the row where staggers start (i), how many rows the planter sows in a pass (j), and the stagger offset from the “A” point (j). For example, if we set “stagger=c(5,4,3.8)” and include two rows of border plots within the input file, plotshpcreate will create a four row stagger (j), 3.8 m towards the back of the field based on the “A” point (k), beginning at fifth row (i) of the field from left to right (**Figure 3A**). The stagger pattern of the field is based on the planter passes, if you have two rows of border and a four row planter, the stagger would begin on the third row of the trial (e.g., stagger=c(3,4,3.8) if border is not included within your input file (**Figure 3B**). If multirow plots are not spilt across planter passes (i.e., there is not staggered adjacent plot rows) the “plotsubset” and “nrowplot” arguments make be implemented in conjunction with “stagger” (**Figures 2E, F**).

## Conclusion

Implementation of high throughput phenotyping platforms such as UAS or ground vehicles can provide a vast amount of data rapidly. In contrast, the development of tools to process sensor datasets is in its infancy, or non-existent, and continued development of data analytic tools is critical to aid rapid data analysis for actionable information extraction (Shakoor et al., 2019). As a result, manual data wrangling remains a laborious time sink in processing sensor datasets. Plotshpcreate was developed to overcome a critical time sink within the data processing pipeline, creating AOIs for research plots at scale. Plotshpcreate provides a tool to rapidly create gridded AOI polygons with attached unique IDs for extraction of sensor data on an agriculture research plot scale within seconds, compared to the hours it would require to manually draw polygons and define unique IDs of thousands of plots within a GIS software. Foundational tools, like plotshpcreate, set the basis for developing more advanced point and click graphical user interface tools, such as shiny (Chang et al., 2019). Additionally, incorporating algorithms that utilize the imagery to auto correct for minor changes in plot orientation (Ribera et al., 2017) would be a useful, although it would likely increase computation time and memory with the inclusion of imagery data analysis. Plotshpcreate has room for improvement through increased functionality and the developers encourage the community to aid in adding new tools and they feel necessary.

## Data Availability Statement

All datasets generated for this study are included in the article/**Supplementary Material**.

## Author Contributions

Conceptualization: SA and SM. Methodology: SA and SM. Software: SA and SM. Validation: SA. Resources: SM. Data Curation: SA. Writing—Original Draft Preparation: SA. Writing—Review and Editing: SA and SM. Visualization: SA. Supervision: SM. Project Administration: SM. Funding Acquisition: SM.

## Funding

This research was funded by USDA-NIFA-AFRI Award No. 2017-67013-26185, USDA-NIFA Hatch funds, Texas A&M AgriLife Research, the Texas Corn Producers Board, the Iowa Corn Promotion Board, and the Eugene Butler Endowed Chair in Biotechnology. SA was funded for one year by the Texas A&M College of Agriculture and Life Sciences Tom Slick Senior Graduate Fellowship. The funders had no involvement in the study. The funder was not involved in the study design, collection, analysis, interpretation of data, the writing of this article, or the decision to submit it for publication.

## Conflict of Interest

The authors declare that the research was conducted in the absence of any commercial or financial relationships that could be construed as a potential conflict of interest.

## Supplementary Material

File S1 R/UAStools v0.2.0 R package.
